# (*E*)-*N*′-(5-Bromo-2-hy­droxy­benzyl­idene)-4-hy­droxy-3-meth­oxy­benzohydrazide methanol solvate

**DOI:** 10.1107/S1600536810022063

**Published:** 2010-06-16

**Authors:** Shi-Yong Liu, Zhonglu You

**Affiliations:** a College of Chemistry & Pharmacy, Taizhou University, Taizhou Zhejiang 317000, People’s Republic of China; b Department of Chemistry, Liaoning Normal University, Dalian 116029, People’s Republic of China

## Abstract

In the title compound, C_15_H_13_BrN_2_O_4_·CH_4_O, the two benzene rings form a dihedral angle of 3.2 (2)°. An intra­molecular O—H⋯N hydrogen bond is observed. In the crystal structure, mol­ecules are linked through O—H⋯O and N—H⋯O hydrogen bonds, forming a two-dimensional network parallel to (10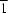).

## Related literature

For the medicinal applications of hydrazone compounds, see: Hillmer *et al.* (2010 ▶); Zhu *et al.* (2009 ▶); Jimenez-Pulido *et al.* (2008 ▶); Raj *et al.* (2007 ▶); Zhong *et al.* (2007 ▶). For crystal structures of hydrazone compounds, see: Khaledi *et al.* (2009 ▶); Warad *et al.* (2009 ▶); Back *et al.* (2009 ▶); Vijayakumar *et al.* (2009 ▶). For related structures, see: Cao (2009 ▶); Xu *et al.* (2009 ▶); Shafiq *et al.* (2009 ▶).
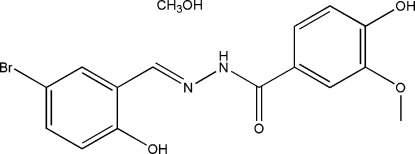


## Experimental

### 

#### Crystal data

C_15_H_13_BrN_2_O_4_·CH_4_O
*M* *_r_* = 397.23
Monoclinic, 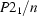

*a* = 7.4412 (4) Å
*b* = 17.5287 (9) Å
*c* = 12.5555 (8) Å
β = 91.200 (3)°
*V* = 1637.31 (16) Å^3^
*Z* = 4
Mo *K*α radiation
μ = 2.54 mm^−1^
*T* = 298 K
0.27 × 0.23 × 0.23 mm

#### Data collection

Bruker SMART CCD area-detector diffractometer
Absorption correction: multi-scan (*SADABS*; Bruker, 2001 ▶) *T*_min_ = 0.547, *T*_max_ = 0.593
9411 measured reflections
3368 independent reflections
2230 reflections with *I* > 2σ(*I*)
*R*_int_ = 0.039

#### Refinement

*R*[*F* ^2^ > 2σ(*F* ^2^)] = 0.036
*wR*(*F* ^2^) = 0.082
*S* = 1.01
3368 reflections
225 parameters
1 restraint
H atoms treated by a mixture of independent and constrained refinement
Δρ_max_ = 0.24 e Å^−3^
Δρ_min_ = −0.31 e Å^−3^

### 

Data collection: *SMART* (Bruker, 2007 ▶); cell refinement: *SAINT* (Bruker, 2007 ▶); data reduction: *SAINT*; program(s) used to solve structure: *SHELXTL* (Sheldrick, 2008 ▶); program(s) used to refine structure: *SHELXTL*; molecular graphics: *SHELXTL*; software used to prepare material for publication: *SHELXTL*.

## Supplementary Material

Crystal structure: contains datablocks global, I. DOI: 10.1107/S1600536810022063/ci5098sup1.cif

Structure factors: contains datablocks I. DOI: 10.1107/S1600536810022063/ci5098Isup2.hkl

Additional supplementary materials:  crystallographic information; 3D view; checkCIF report

## Figures and Tables

**Table 1:** Hydrogen-bond geometry (Å, °)

*D*—H⋯*A*	*D*—H	H⋯*A*	*D*⋯*A*	*D*—H⋯*A*
N2—H2⋯O5	0.89 (1)	2.07 (1)	2.949 (3)	167 (3)
O1—H1⋯N1	0.82	1.98	2.686 (3)	145
O4—H4⋯O2^i^	0.82	1.87	2.671 (3)	166
O5—H5⋯O3^ii^	0.82	2.46	3.125 (3)	139
O5—H5⋯O4^ii^	0.82	2.57	3.252 (3)	141

Symmetry codes: (i) 
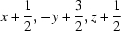
; (ii) 
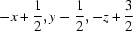
.

**Fig. 1.**
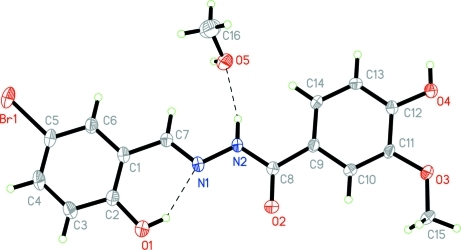
The molecular structure of the title compound. Displacement ellipsoids are drawn at the 30% probability level. Hydrogen atoms are shown as spheres of arbitrary radius. Hydrogen bonds are shown as dashed lines.

**Fig. 2.**
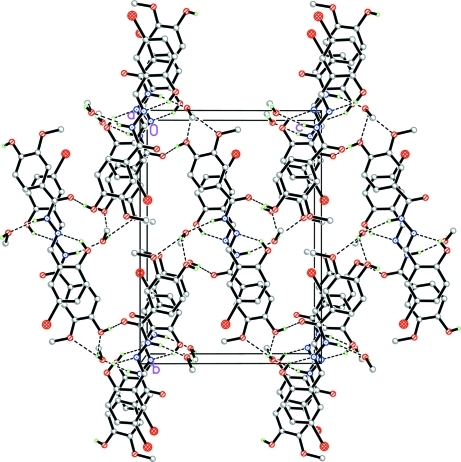
The molecular packing of the title compound, viewed along the a axis. Hydrogen bonds are shown as dashed lines. Hydrogen atoms not involved in hydrogen bonding have been omitted.

## supplementary crystallographic information

### Comment

Considerable attention has been focused on hydrazones and their medicinal applications (Hillmer *et al.*, 2010; Zhu *et al.*, 2009; Jimenez-Pulido *et al.*, 2008; Raj *et al.*, 2007; Zhong *et al.*, 2007). The study on the crystal structures of such compounds is of particular interest (Khaledi *et al.*, 2009; Warad *et al.*, 2009; Back *et al.*, 2009; Vijayakumar *et al.*, 2009). We report herein the crystal structure of the title new hydrazone.

The asymmetric unit of the title compound contains a benzohydrazide molecule and a methanol solvate molecule, as shown in Fig. 1. The dihedral angle between the two benzene rings is 3.2 (2)°, indicating they are nearly coplanar. Atom C15 deviates from the C9–C14 benzene ring by 0.188 (2) Å. All the bond lengths are comparable to those observed in related structures (Cao, 2009; Xu *et al.*, 2009; Shafiq *et al.*, 2009).

In the crystal structure, the hydrazone and methanol molecules are linked through O—H···N, O—H···O, and N—H···O hydrogen bonds, to form a two-dimensional network parallel to the (101) (Fig. 2 and Table 1).

### Experimental

The title compound was prepared by the condensation reaction of 5-bromosalicylaldehyde (0.05 mol, 10 g) and 4-hydroxy-3-methoxybenzohydrazide (0.05 mol, 9 g) in anhydrous methanol (200 ml) at ambient temperature. Colourless block-shaped single crystals suitable for X-ray structural determination were obtained by slow evaporation of the methanol solution for a period of 5 d.

### Refinement

Atom H2 was located in a difference map and refined isotropically, with the N–H distance restrained to 0.90 (1) Å; U_iso_(H2) was fixed to 0.08 Å^2^. The remaining H atoms were positioned geometrically and constrained to ride on their parent atoms, with C–H distances of 0.93–0.96 Å, O–H  distances of 0.82 Å, and with *U*_iso_(H) = 1.2*U*_eq_(C) and 1.5*U*_eq_(C_methyl_ and O).

### Crystal data

**Table:**

C_15_H_13_BrN_2_O_4_·CH_4_O	*F*(000) = 808
*M**_r_* = 397.23	*D*_x_ = 1.611 Mg m^−^^3^
Monoclinic, *P*2_1_/*n*	Mo *K*α radiation, λ = 0.71073 Å
Hall symbol: -P 2yn	Cell parameters from 2105 reflections
*a* = 7.4412 (4) Å	θ = 2.3–24.0°
*b* = 17.5287 (9) Å	µ = 2.54 mm^−^^1^
*c* = 12.5555 (8) Å	*T* = 298 K
β = 91.200 (3)°	Block, colourless
*V* = 1637.31 (16) Å^3^	0.27 × 0.23 × 0.23 mm
*Z* = 4	

### Data collection

**Table:**

Bruker SMART CCD area-detector diffractometer	3368 independent reflections
Radiation source: fine-focus sealed tube	2230 reflections with *I* > 2σ(*I*)
graphite	*R*_int_ = 0.039
ω scans	θ_max_ = 26.6°, θ_min_ = 2.0°
Absorption correction: multi-scan (*SADABS*; Bruker, 2001)	*h* = −9→9
*T*_min_ = 0.547, *T*_max_ = 0.593	*k* = −19→21
9411 measured reflections	*l* = −13→15

### Refinement

**Table:**

Refinement on *F*^2^	Primary atom site location: structure-invariant direct methods
Least-squares matrix: full	Secondary atom site location: difference Fourier map
*R*[*F*^2^ > 2σ(*F*^2^)] = 0.036	Hydrogen site location: inferred from neighbouring sites
*wR*(*F*^2^) = 0.082	H atoms treated by a mixture of independent and constrained refinement
*S* = 1.01	*w* = 1/[σ^2^(*F*_o_^2^) + (0.0375*P*)^2^] where *P* = (*F*_o_^2^ + 2*F*_c_^2^)/3
3368 reflections	(Δ/σ)_max_ = 0.001
225 parameters	Δρ_max_ = 0.24 e Å^−^^3^
1 restraint	Δρ_min_ = −0.31 e Å^−^^3^

### Special details

**Table:**

Geometry. All esds (except the esd in the dihedral angle between two l.s. planes) are estimated using the full covariance matrix. The cell esds are taken into account individually in the estimation of esds in distances, angles and torsion angles; correlations between esds in cell parameters are only used when they are defined by crystal symmetry. An approximate (isotropic) treatment of cell esds is used for estimating esds involving l.s. planes.
Refinement. Refinement of F^2^ against ALL reflections. The weighted R-factor wR and goodness of fit S are based on F^2^, conventional R-factors R are based on F, with F set to zero for negative F^2^. The threshold expression of F^2^ > 2sigma(F^2^) is used only for calculating R-factors(gt) etc. and is not relevant to the choice of reflections for refinement. R-factors based on F^2^ are statistically about twice as large as those based on F, and R- factors based on ALL data will be even larger.

### Fractional atomic coordinates and isotropic or equivalent isotropic displacement parameters (Å^2^)

**Table:**

	*x*	*y*	*z*	*U*_iso_*/*U*_eq_	
Br1	0.18595 (5)	0.149648 (15)	0.52921 (3)	0.05687 (14)	
N1	0.2299 (3)	0.51490 (11)	0.46909 (16)	0.0362 (5)	
N2	0.2800 (3)	0.57506 (11)	0.53449 (16)	0.0370 (5)	
O1	0.0923 (3)	0.44648 (10)	0.29416 (14)	0.0550 (6)	
H1	0.1171	0.4831	0.3324	0.083*	
O2	0.2525 (3)	0.65966 (10)	0.40042 (15)	0.0508 (5)	
O3	0.3700 (3)	0.91862 (9)	0.56842 (13)	0.0438 (5)	
O4	0.5122 (3)	0.89035 (10)	0.75563 (14)	0.0513 (5)	
H4	0.5720	0.8758	0.8073	0.077*	
O5	0.3071 (3)	0.51702 (11)	0.75469 (16)	0.0561 (6)	
H5	0.2163	0.4979	0.7793	0.084*	
C1	0.1853 (4)	0.38040 (14)	0.4545 (2)	0.0363 (6)	
C2	0.1173 (4)	0.38095 (15)	0.3500 (2)	0.0382 (6)	
C3	0.0704 (4)	0.31294 (16)	0.3002 (2)	0.0468 (7)	
H3	0.0236	0.3136	0.2309	0.056*	
C4	0.0928 (4)	0.24469 (15)	0.3524 (2)	0.0489 (8)	
H4A	0.0631	0.1992	0.3182	0.059*	
C5	0.1593 (4)	0.24389 (14)	0.4555 (2)	0.0408 (7)	
C6	0.2052 (4)	0.31014 (14)	0.5065 (2)	0.0393 (7)	
H6	0.2498	0.3086	0.5763	0.047*	
C7	0.2359 (4)	0.44864 (14)	0.5118 (2)	0.0386 (7)	
H7	0.2743	0.4443	0.5824	0.046*	
C8	0.2886 (3)	0.64661 (13)	0.4938 (2)	0.0329 (6)	
C9	0.3473 (3)	0.70777 (13)	0.56902 (19)	0.0305 (6)	
C10	0.3292 (3)	0.78280 (13)	0.5325 (2)	0.0328 (6)	
H10	0.2810	0.7921	0.4648	0.039*	
C11	0.3827 (3)	0.84311 (12)	0.5964 (2)	0.0317 (6)	
C12	0.4587 (4)	0.82893 (14)	0.69653 (19)	0.0336 (6)	
C13	0.4745 (4)	0.75503 (13)	0.7325 (2)	0.0357 (6)	
H13	0.5227	0.7457	0.8002	0.043*	
C14	0.4193 (3)	0.69455 (14)	0.66906 (19)	0.0350 (6)	
H14	0.4310	0.6448	0.6941	0.042*	
C15	0.3218 (4)	0.93558 (16)	0.4607 (2)	0.0530 (8)	
H15A	0.4045	0.9110	0.4141	0.079*	
H15B	0.3261	0.9898	0.4498	0.079*	
H15C	0.2022	0.9174	0.4455	0.079*	
C16	0.4575 (5)	0.4742 (2)	0.7866 (3)	0.0815 (11)	
H16A	0.5649	0.5019	0.7707	0.122*	
H16B	0.4572	0.4264	0.7491	0.122*	
H16C	0.4536	0.4648	0.8618	0.122*	
H2	0.291 (4)	0.5653 (18)	0.6041 (10)	0.080*	

### Atomic displacement parameters (Å^2^)

**Table:**

	*U*^11^	*U*^22^	*U*^33^	*U*^12^	*U*^13^	*U*^23^
Br1	0.0671 (2)	0.02604 (16)	0.0779 (3)	−0.00003 (14)	0.01145 (17)	0.00364 (15)
N1	0.0442 (14)	0.0265 (12)	0.0378 (13)	−0.0026 (10)	−0.0020 (10)	−0.0059 (10)
N2	0.0540 (15)	0.0219 (11)	0.0346 (12)	−0.0025 (10)	−0.0071 (12)	−0.0032 (10)
O1	0.0824 (16)	0.0330 (11)	0.0492 (12)	−0.0019 (11)	−0.0106 (11)	0.0004 (9)
O2	0.0814 (16)	0.0344 (11)	0.0359 (11)	−0.0045 (10)	−0.0189 (10)	0.0029 (8)
O3	0.0658 (14)	0.0215 (9)	0.0437 (11)	0.0019 (8)	−0.0123 (10)	0.0040 (8)
O4	0.0815 (17)	0.0257 (10)	0.0458 (13)	0.0000 (9)	−0.0218 (11)	−0.0052 (8)
O5	0.0667 (15)	0.0455 (12)	0.0558 (13)	−0.0083 (11)	−0.0048 (12)	0.0131 (10)
C1	0.0394 (17)	0.0283 (13)	0.0413 (16)	−0.0033 (12)	0.0036 (13)	−0.0056 (12)
C2	0.0431 (17)	0.0291 (13)	0.0424 (16)	−0.0010 (12)	0.0031 (13)	−0.0013 (12)
C3	0.052 (2)	0.0403 (17)	0.0474 (17)	−0.0067 (14)	−0.0019 (14)	−0.0107 (14)
C4	0.057 (2)	0.0309 (15)	0.059 (2)	−0.0117 (13)	0.0078 (16)	−0.0125 (14)
C5	0.0424 (17)	0.0249 (14)	0.0554 (19)	−0.0001 (11)	0.0095 (14)	−0.0025 (13)
C6	0.0464 (18)	0.0289 (14)	0.0428 (16)	−0.0031 (12)	0.0026 (14)	−0.0021 (12)
C7	0.0520 (18)	0.0277 (14)	0.0358 (15)	−0.0001 (12)	−0.0031 (13)	−0.0012 (12)
C8	0.0380 (16)	0.0268 (13)	0.0338 (15)	−0.0014 (11)	−0.0027 (12)	0.0006 (11)
C9	0.0362 (16)	0.0212 (12)	0.0341 (14)	−0.0007 (10)	0.0015 (12)	0.0009 (10)
C10	0.0365 (16)	0.0285 (13)	0.0331 (14)	0.0023 (11)	−0.0051 (12)	0.0039 (11)
C11	0.0375 (16)	0.0179 (12)	0.0396 (15)	0.0030 (10)	−0.0003 (12)	0.0015 (11)
C12	0.0395 (16)	0.0267 (13)	0.0343 (15)	−0.0007 (11)	−0.0030 (12)	−0.0031 (11)
C13	0.0483 (18)	0.0307 (14)	0.0277 (13)	−0.0004 (12)	−0.0081 (12)	0.0018 (11)
C14	0.0455 (17)	0.0242 (13)	0.0352 (15)	−0.0015 (11)	−0.0032 (13)	0.0059 (11)
C15	0.077 (2)	0.0306 (15)	0.0507 (19)	−0.0022 (15)	−0.0197 (16)	0.0146 (13)
C16	0.080 (3)	0.077 (3)	0.087 (3)	0.003 (2)	−0.015 (2)	0.018 (2)

### Geometric parameters (Å, °)

**Table:**

Br1—C5	1.902 (3)	C4—H4A	0.93
N1—C7	1.279 (3)	C5—C6	1.367 (3)
N1—N2	1.383 (3)	C6—H6	0.93
N2—C8	1.356 (3)	C7—H7	0.93
N2—H2	0.893 (10)	C8—C9	1.489 (3)
O1—C2	1.357 (3)	C9—C14	1.375 (3)
O1—H1	0.82	C9—C10	1.399 (3)
O2—C8	1.218 (3)	C10—C11	1.381 (3)
O3—C11	1.372 (3)	C10—H10	0.93
O3—C15	1.423 (3)	C11—C12	1.390 (3)
O4—C12	1.362 (3)	C12—C13	1.376 (3)
O4—H4	0.82	C13—C14	1.383 (3)
O5—C16	1.400 (4)	C13—H13	0.93
O5—H5	0.82	C14—H14	0.93
C1—C2	1.395 (4)	C15—H15A	0.96
C1—C6	1.401 (4)	C15—H15B	0.96
C1—C7	1.442 (3)	C15—H15C	0.96
C2—C3	1.388 (4)	C16—H16A	0.96
C3—C4	1.373 (4)	C16—H16B	0.96
C3—H3	0.93	C16—H16C	0.96
C4—C5	1.376 (4)		
			
C7—N1—N2	115.9 (2)	N2—C8—C9	116.2 (2)
C8—N2—N1	119.7 (2)	C14—C9—C10	119.4 (2)
C8—N2—H2	123 (2)	C14—C9—C8	124.2 (2)
N1—N2—H2	117 (2)	C10—C9—C8	116.4 (2)
C2—O1—H1	109.5	C11—C10—C9	120.3 (2)
C11—O3—C15	117.37 (19)	C11—C10—H10	119.9
C12—O4—H4	109.5	C9—C10—H10	119.9
C16—O5—H5	109.5	O3—C11—C10	124.9 (2)
C2—C1—C6	118.5 (2)	O3—C11—C12	115.4 (2)
C2—C1—C7	123.3 (2)	C10—C11—C12	119.7 (2)
C6—C1—C7	118.2 (2)	O4—C12—C13	122.9 (2)
O1—C2—C3	117.6 (2)	O4—C12—C11	117.4 (2)
O1—C2—C1	122.3 (2)	C13—C12—C11	119.7 (2)
C3—C2—C1	120.0 (3)	C12—C13—C14	120.6 (2)
C4—C3—C2	120.4 (3)	C12—C13—H13	119.7
C4—C3—H3	119.8	C14—C13—H13	119.7
C2—C3—H3	119.8	C9—C14—C13	120.2 (2)
C3—C4—C5	119.7 (3)	C9—C14—H14	119.9
C3—C4—H4A	120.1	C13—C14—H14	119.9
C5—C4—H4A	120.1	O3—C15—H15A	109.5
C6—C5—C4	120.9 (3)	O3—C15—H15B	109.5
C6—C5—Br1	119.1 (2)	H15A—C15—H15B	109.5
C4—C5—Br1	119.9 (2)	O3—C15—H15C	109.5
C5—C6—C1	120.3 (2)	H15A—C15—H15C	109.5
C5—C6—H6	119.8	H15B—C15—H15C	109.5
C1—C6—H6	119.8	O5—C16—H16A	109.5
N1—C7—C1	122.5 (2)	O5—C16—H16B	109.5
N1—C7—H7	118.7	H16A—C16—H16B	109.5
C1—C7—H7	118.7	O5—C16—H16C	109.5
O2—C8—N2	121.7 (2)	H16A—C16—H16C	109.5
O2—C8—C9	122.1 (2)	H16B—C16—H16C	109.5

### Hydrogen-bond geometry (Å, °)

**Table:**

*D*—H···*A*	*D*—H	H···*A*	*D*···*A*	*D*—H···*A*
N2—H2···O5	0.89 (1)	2.07 (1)	2.949 (3)	167 (3)
O1—H1···N1	0.82	1.98	2.686 (3)	145
O4—H4···O2^i^	0.82	1.87	2.671 (3)	166
O5—H5···O3^ii^	0.82	2.46	3.125 (3)	139
O5—H5···O4^ii^	0.82	2.57	3.252 (3)	141

Symmetry codes: (i) *x*+1/2, −*y*+3/2, *z*+1/2; (ii) −*x*+1/2, *y*−1/2, −*z*+3/2.
